# Anti-Inflammatory Effects of a *Cordyceps sinensis* Mycelium Culture Extract (Cs-4) on Rodent Models of Allergic Rhinitis and Asthma

**DOI:** 10.3390/molecules25184051

**Published:** 2020-09-04

**Authors:** Jihang Chen, Wing Man Chan, Hoi Yan Leung, Pou Kuan Leong, Choly Tat Ming Yan, Kam Ming Ko

**Affiliations:** 1 School of Life and Health Science, The Chinese University of Hong Kong, Shenzhen 518172, China; chenjihang2008@gmail.com; 2 Division of Life Science, The Hong Kong University of Science & Technology, Clear Water Bay, Hong Kong SAR 999077, China; wingman@ust.hk (W.M.C.); hoiyan@ust.hk (H.Y.L.); bc_lpkaa@alumni.ust.hk (P.K.L.); 3 Royal Medic Group Holding Limited, 313 Castle Peak Road, Hong Kong SAR 999077, China; cholyyan@gmail.com

**Keywords:** Cordyceps, anti-inflammatory effect, allergic rhinitis, asthma

## Abstract

Allergic rhinitis and asthma are common chronic allergic diseases of the respiratory tract, which are accompanied by immunoglobulin E (IgE)-mediated inflammation and the involvement of type 2 T helper cells, mast cells, and eosinophils. *Cordyceps sinensis* (Berk.) Sacc is a fungal parasite on the larva of Lepidoptera. It has been considered to be a health-promoting food and, also, one of the best-known herbal remedies for the treatment of airway diseases, such as asthma and lung inflammation. In the present study, we demonstrated the antiallergic rhinitis effect of Cs-4, a water extract prepared from the mycelium culture of *Cordyceps sinensis* (Berk) Sacc, on ovalbumin (OVA)-induced allergic rhinitis in mice and the anti-asthmatic effect of Cs-4 in a rat model of asthma. Treatment with Cs-4 suppressed the nasal symptoms induced in OVA-sensitized and challenged mice. The inhibition was associated with a reduction in IgE/OVA-IgE and interleukin (IL)-4/IL-13 levels in the nasal fluid. Cs-4 treatment also decreased airway responsiveness and ameliorated the scratching behavior in capsaicin-challenged rats. It also reduced plasma IgE levels, as well as IgE and eosinophil peroxidase levels, in the bronchoalveolar fluid. Cs-4 treatment completely suppressed the increases in IL-4, IL-5, and IL-13 levels in rat lung tissue. In conclusion, our results suggest that Cs-4 has the potential to alleviate immune hypersensitivity reactions in allergic rhinitis and asthma.

## 1. Introduction

Allergic rhinitis (AR) and asthma are common chronic allergic diseases of the respiratory tract. The prevalence of AR and asthma has been estimated to range from 3% to 19% and 4%, respectively, globally [1]. Patients with AR present with one or more of the following symptoms: nasal congestion, rhinorrhea, sneezing, and itching [2]. Asthma is characterized by respiratory airway symptoms, including wheezing, coughing, shortness of breath, and a feeling of tightness in the chest [3]. As the upper respiratory mucosa is continuous with the mucosa of lower airways, up to 40% of asthmatic patients show symptoms of AR [4]. By the same token, patients with AR are likely to develop bronchial asthma three times more frequently than non-rhinitis patients [4].

AR and asthma involve complex pathological processes, which are characterized by immunoglobulin E (IgE)-mediated inflammation and the involvement of type 2 T-helper cells (Th2), mast cells, and eosinophils [5]. There are two phases in an allergic reaction—namely, early and late [6]. The early phase involves the crosslinking of allergen and allergen-specific IgE receptors on the surface of mast cells, resulting in the release of histamine and an increase in vascular permeability [7]. This is followed by late-phase reactions, which involve the mediator-induced recruitment of more inflammatory cells, such as eosinophils, neutrophils, macrophages, and lymphocytes [8]. This infiltration of leukocytes to the site of involvement leads to a decrease in the airway diameter and mucus secretion, resulting in the manifestation of nasal and bronchial symptoms [5].

*Cordyceps sinensis* (Berk.) Sacc is a fungal parasite that grows on the larva of Lepidoptera. It is considered to be a health-promoting product and, also, one of the best-known herbal remedies for the treatment of airway diseases, such as asthma and lung inflammation [9]. *Cordyceps sinensis* with its parasitic hosts has a harsh growing environment in the wild, and the selling price (up to US 30 per gram, cf. Amazon website) is soaring due to the recent elucidation of the pharmacological basis of its health-promoting effects [9]. Currently, Cordyceps mycelium is cultivated by a process involving deep fermentation and has been widely used as a substitute for *Cordyceps sinensis* in medicines or herbal products [10]. *Cordyceps sinensis* extracts have been reported to possess anti-inflammatory and immunomodulatory activities [11]. Such extracts have also been shown to inhibit airway hyperresponsiveness and eosinophil infiltration in rat models of allergic responses [12]. While anti-asthmatic activities produced by *Cordyceps splecophala* mycelium extract have been reported in mice [13], whether or not the administration of a *Cordyceps sinensis* mycelium extract can exert anti-AR and anti-asthmatic effects remains largely unknown. In the present study, we aimed to investigate: (1) the antiallergic rhinitis effect of Cs-4, a water extract prepared from mycelium cultures of *Cordyceps sinensis* (Berk) Sacc, on ovalbumin (OVA)-induced allergic rhinitis in mice and (2) the anti-asthmatic effect of Cs-4 in a rat model of asthma.

## 2. Results

### 2.1. Marker Compound Contents in Cs-4

The contents of adenine, adenosine, and cordycepin were found to be 0.038%, 0.255%, and <0.013% (*w/w*), respectively.

### 2.2. Cs-4 Treatment Suppressed the OVA Challenge Produced Nasal Symptoms in OVA-Sensitized Mice

**Figure 1:**
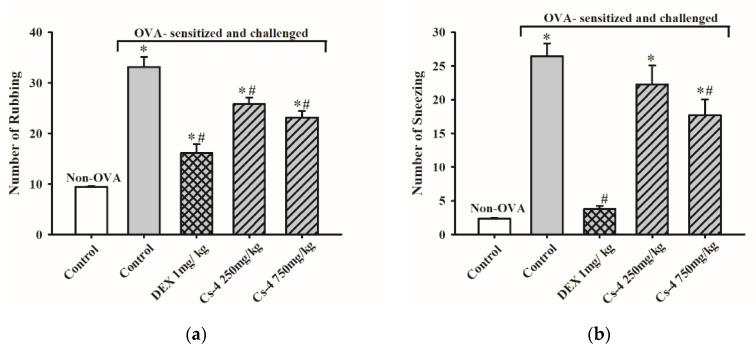
The effect of Cs-4 on the nasal symptoms of ovalbumin (OVA)-sensitized and challenged mice. Nasal symptoms of (**a**) nose rubbing and (**b**) sneezing were observed. Each bar represents the mean ± S.E.M., with *n* = 5. * Significantly different from the non-OVA control group. # Significantly different from the OVA-sensitized and challenged control group. DEX: dexamethasone.

As shown in Figure 1, the OVA challenge produced nasal symptoms in OVA-sensitized mice, as evidenced by increases in the number of nose rubbings (2.5-fold) and sneezing (10.2-fold), when compared with the unsensitized and unchallenged controls. Dexamethasone (DEX), a clinically used steroidal anti-inflammatory drug, was used as a positive control. DEX treatment (1 mg/kg, i.g. (intragastrically)) largely and significantly suppressed the frequency of nose rubbing and sneezing by 72 and 94%, respectively, when compared with the untreated, OVA-sensitized, and challenged controls. Cs-4 treatment at 250 or 750 mg/kg significantly reduced the frequency of nose rubbing by 31 and 58%, respectively, when compared with the untreated, OVA-sensitized, and challenged controls. The frequency of sneezing was also significantly decreased (63%) by Cs-4 treatment at a dose of 0.75 g/kg.

### 2.3. Cs-4 Treatment Reduced the Levels of IgE and OVA-specific IgE in the Lavage Fluid (NFL) of OVA-Sensitized and Challenged Mice

**Figure 2:**
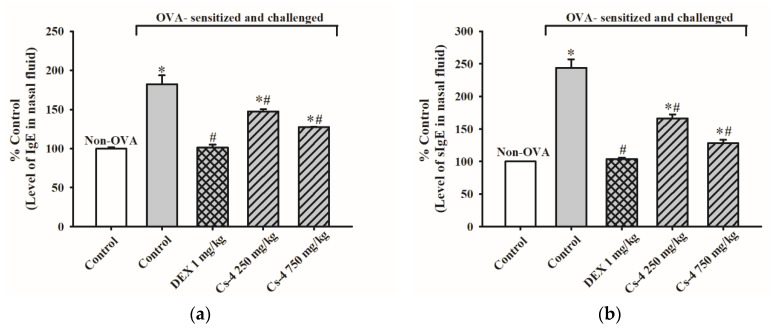
The effect of Cs-4 on (**a**) the levels of immunoglobulin E (IgE) and (**b**) OVA-specific IgE in the lavage nasal fluid (NFL) of OVA-sensitized and challenged mice. Each bar represents the mean ± S.E.M., with *n* = 5. * Significantly different from the non-OVA control group. # Significantly different from the OVA-sensitized and challenged control group.

Figure 2 shows the effects of Cs-4 on the levels of IgE and OVA-specific IgE in the nasal fluid of OVA-sensitized and challenged mice. The OVA challenge induced significant increases in both IgE (82%) and OVA-specific IgE (144%) in the nasal fluid of OVA-sensitized mice. The DEX treatment (1 mg/kg, i.g.) caused a complete suppression of OVA-induced elevations in the IgE and OVA-specific IgE levels. Cs-4 (250 and 750 mg/kg) significantly reduced the levels of IgE (42 and 67%) and OVA-specific IgE (54 and 80%) in OVA-sensitized and challenged mice, when compared with the untreated, sensitized, and challenged controls.

### 2.4. Cs-4 Treatment Significantly Decreased both IL-4 and IL-13 Levels in the NLF of OVA-Sensitized and Challenged Mice

**Figure 3:**
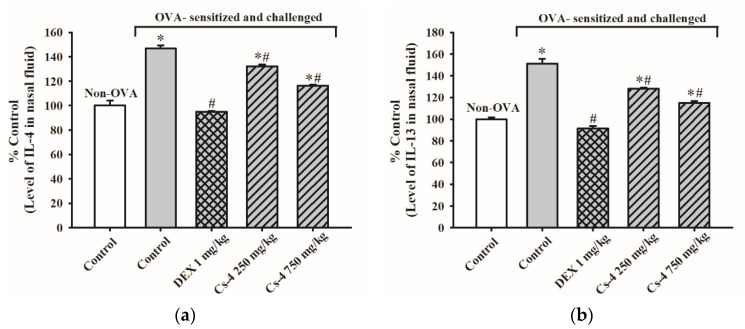
The effect of Cs-4 on (**a**) the levels of interleukin (IL)-4 and (**b**) IL-13 in the NLF of OVA-sensitized and challenged mice. Each bar represents the mean ± S.E.M., with *n* = 5. * Significantly different from the non-OVA control group. # Significantly different from the OVA-sensitized and challenged control group.

As shown in Figure 3, the OVA challenge caused increases in the IL-4 (47%) and IL-13 (51%) levels in the NFL of OVA-sensitized mice, when compared with the unsensitized and challenged controls. The DEX treatment (1 mg/kg, i.g.) completely abrogated the OVA-induced elevation of IL-4 and IL-13 levels in the NLF of OVA-sensitized mice. The Cs-4 treatment (250 and 750 mg/kg) significantly decreased both IL-4 (31 and 65%) and IL-13 (45 and 70%) levels in the NLF of OVA-sensitized and challenged mice, when compared with the untreated, OVA-sensitized, and challenged controls.

### 2.5. Preincubation with Cs-4 Suppressed C48/80-Activated β-Hexosaminidase Release

**Figure 4:**
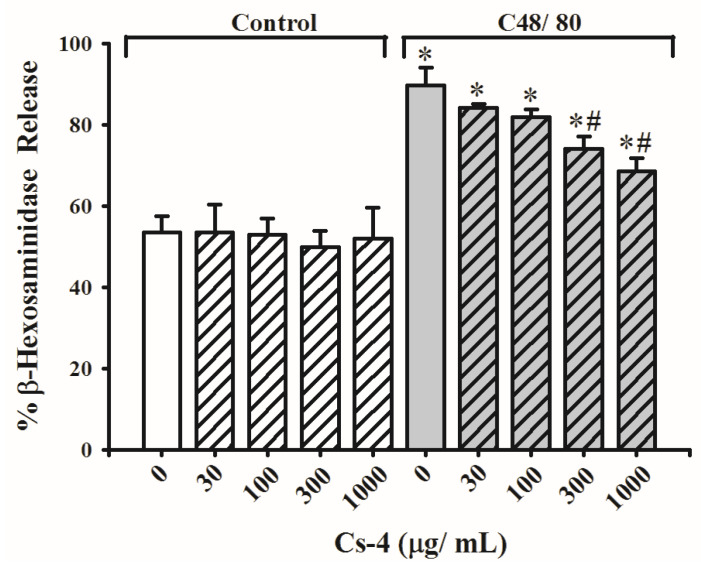
The effects of Cs-4 on the C48/80-activated β-hexosaminidase release in rat peritoneal mast cells (RPMC) in vitro. Each bar represents the mean ± SD, with *n* = 6. * Significantly different from the control group. # Significantly different from the C48/80 non-Cs-4-incubated control group.

Figure 4 shows the effects of Cs-4 on C48/80-activated β-hexosaminidase releases in rat peritoneal mast cells (RPMC) in vitro. While preincubation with increasing concentrations (30–1000 µg/mL) of Cs-4 did not affect β-hexosaminidase release in unactivated RPMC, C48/80 activation significantly increased β-hexosaminidase release by 67%, when compared with the unstimulated controls. Preincubation with Cs-4 significantly suppressed C48/80-activated β-hexosaminidase release at concentrations of 300 and 1000 µg/mL by 43 and 58%, respectively, when compared with activated and non-Cs-4 preincubated controls.

### 2.6. Preincubation with Cs-4 Suppressed C48/80-Activated Histamine Release

**Figure 5:**
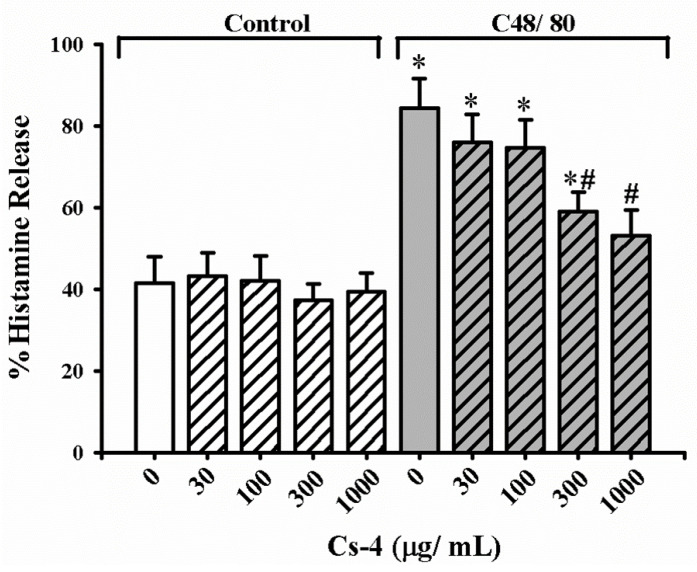
The effects of Cs-4 on the C48/80-activated histamine release in rat RPMC in vitro. Each bar represents the mean ± SD, with *n* = 6. * Significantly different from the control group. # Significantly different from the C48/80 non-Cs-4-incubated control group.

Figure 5 shows the effects of Cs-4 on the C48/80-activated histamine release in RPMC in vitro. While preincubation with increasing concentrations (30–1000 µg/mL) of Cs-4 did not affect the histamine release in unactivated RPMC, C48/80 activation significantly increased the histamine release by 103%, when compared with the unstimulated controls. Preincubation with Cs-4 significantly suppressed the C48/80-activated histamine release by 59 and 73%, respectively, at concentrations of 300 and 1000 µg/mL, when compared with the activated and non-Cs-4 preincubated controls.

### 2.7. Cs-4 Treatment Did Not Produce any Detectable Change in the Body Weight of Capsaicin-Challenged Rats, but the Splenic Index Was Reduced When Compared with the Challenged and Untreated Controls

Table 1 shows the effects of Cs-4 on the body weight and splenic index of capsaicin-challenged rats. Whereas the neonatal capsaicin challenge did not affect the body weights of rats 46 days after birth, the splenic index was significantly increased by 20%, when compared with the nonchallenged controls. The DEX treatment (1 mg/kg) beginning after weaning significantly reduced the body weight (61%) and splenic index (34%) of capsaicin-challenged rats, when compared with the untreated and challenged controls. Although the Cs-4 treatments (123 and 375 mg/kg) did not produce any detectable changes in the body weights of capsaicin-challenged rats, the splenic index was significantly reduced by 78% at a dose of 0.375 g/kg, when compared with the challenged and untreated controls.

### 2.8. Cs-4 Treatment Lowered the Cutaneous Lesion Induced by the Capsaicin Challenge on Rats

The capsaicin challenge significantly increased the cutaneous lesion score by 19.7-fold in rats. The DEX treatment (1 mg/kg) slightly but insignificantly decreased cutaneous lesion scores in capsaicin-challenged rats. The Cs-4 treatment at a dose of 375 mg/kg significantly lowered the score by 73% (Table 2).

### 2.9. Cs-4 Treatment Decreased the EC_50_ of Methacholine (Mch) on the Induction of Contractions on Tracheal Rings and Bronchial Rings

**Figure 6:**
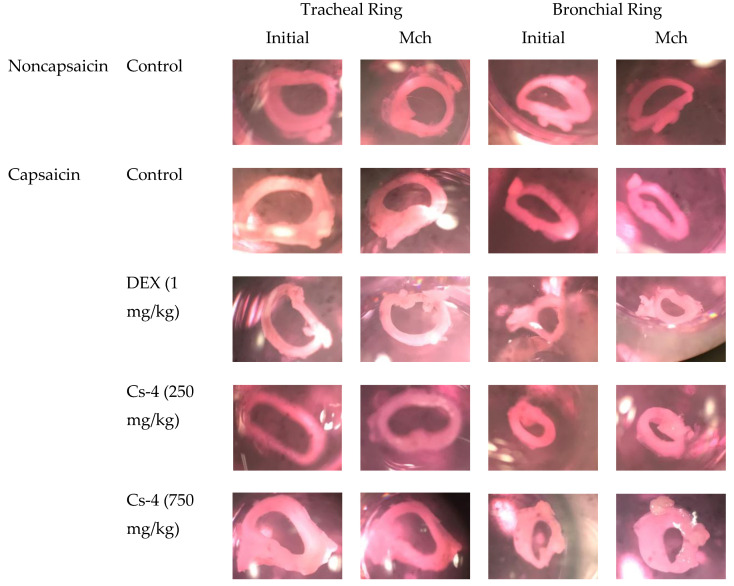
Representative images of the tracheal or bronchial rings taken before (initial) and after 1-μM methacholine (Mch) was added.

Table 3 and Figure 6 show the effect of Cs-4 on airway (trachea and bronchus) responsiveness in capsaicin-challenged rats. The capsaicin challenge increased the responsiveness of both tracheal and bronchial rings to Mch-induced contractions, as evidenced by significant increases in the effective concentration (EC_50_) by 99 and 96%, respectively, when compared with the unchallenged controls. The DEX treatment (1 mg/kg) did not produce any detectable effects on the airway responsiveness in capsaicin-challenged rats. The Cs-4 treatment (0.123 and 0.375 g/kg) significantly decreased the EC_50_ of the tracheal rings (47 and 88%) and bronchial rings (158 and 228%), when compared with the challenged and untreated controls.

### 2.10. Cs-4 Treatment Reduced the Number of Scratching Events

**Figure 7:**
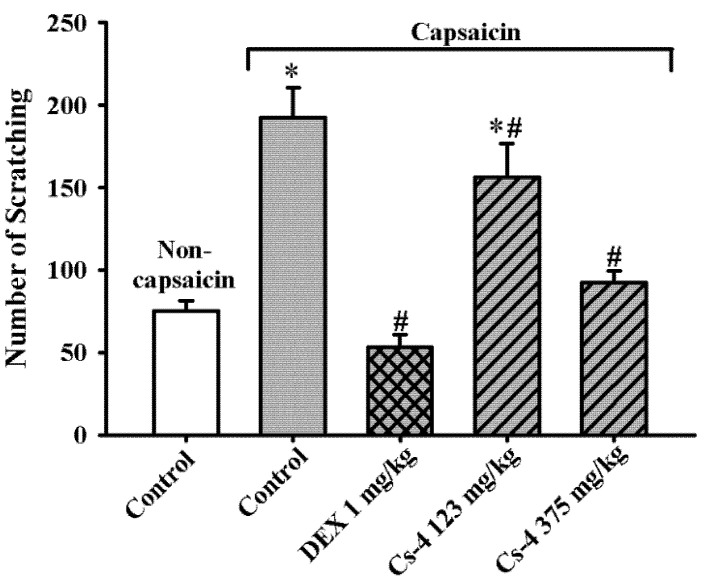
The effect of Cs-4 on the scratching behavior of capsaicin-challenged rats. The rat model of asthma was established, and scratching behavior was scored as described in the Materials and Methods. Each bar represents the mean ± S.E.M., with *n* = 8. * Significantly different from the noncapsaicin control group. # Significantly different from the capsaicin control group.

As shown in Figure 7, the capsaicin challenge triggered scratching behavior in rats, as evidenced by a significant increase (156%) in scratching events, when compared with the unchallenged controls. The DEX treatment (1 mg/kg) completely suppressed the capsaicin-induced stimulation of scratching behavior. The Cs-4 treatment (123 and 375 mg/kg) significantly reduced the number of scratching events by 31 and 85%, respectively, when compared with the challenged and untreated controls.

### 2.11. Cs-4 Treatment Reduced the Plasma IgE Levels in Capsaicin-Challenged Rats

**Figure 8:**
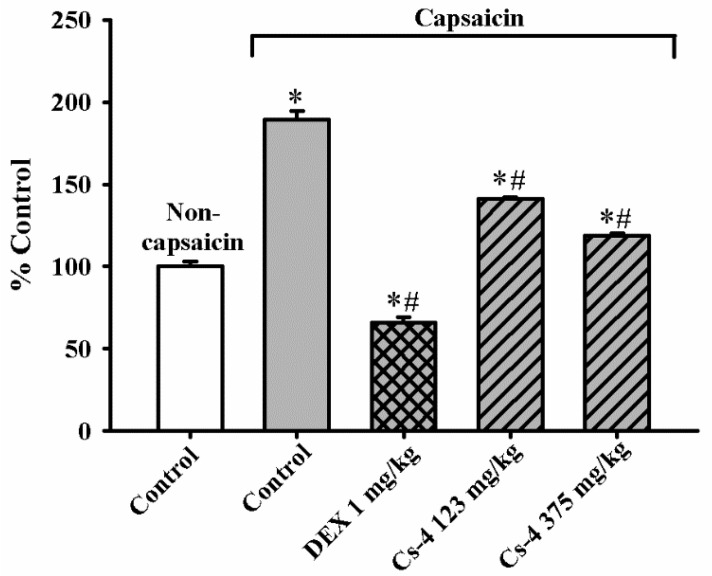
The effect of Cs-4 on the plasma IgE levels in capsaicin-challenged rats. Data were expressed as the control percent of noncapsaicin rats, with the control value being 16.9 ± 0.47 ng/mL. Each bar represents the mean ± S.E.M., with *n* = 8. * Significantly different from the noncapsaicin control group. # Significantly different from the capsaicin control group.

Figure 8 shows the effect of Cs-4 on the plasma IgE levels in capsaicin-challenged rats. The capsaicin challenge caused a significant increase (90%) in the plasma IgE levels in rats. The DEX treatment (1 mg/kg) completely inhibited the capsaicin-induced increase in the plasma IgE levels. The Cs-4 treatment (123 and 375 mg/kg) significantly reduced the plasma IgE levels (54 and 79%) in capsaicin-challenged rats, when compared with the challenged and untreated controls.

### 2.12. Cs-4 Treatment Decreased the IgE and Eosinophil Peroxidase (EPO) Levels in the Bronchoalveolar (BAL) Fluid of Capsaicin-Challenged Rats

**Figure 9:**
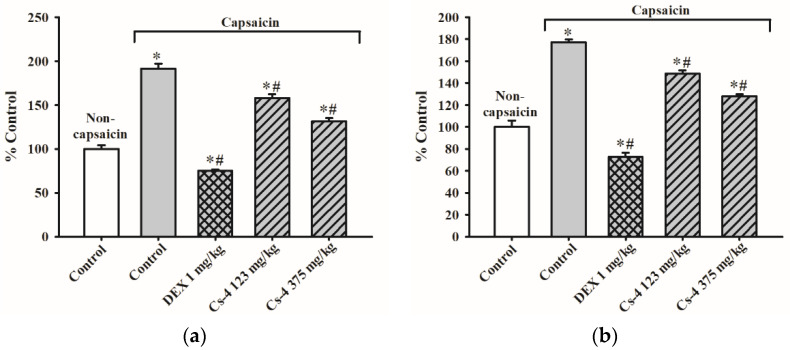
The effect of Cs-4 on the (**a**) IgE level and (**b**) eosinophil peroxidase (EPO) level in the bronchoalveolar (BAL) fluid of capsaicin-challenged rats. Data were expressed as the control percent of noncapsaicin rats, with the control value being (a) 13.3 ± 0.58 ng/mL IgE and (b) 0.600 ± 0.034 ng/mL EPO. Each bar represents the mean ± S.E.M., with *n* = 8. * Significantly different from the noncapsaicin control group. # Significantly different from the capsaicin control group.

As shown in Figure 9, the capsaicin challenge significantly increased the levels of IgE (91%) and EPO (77%) in the BAL fluid in rats, when compared with the unchallenged controls. The DEX treatment (1 mg/kg) completely abolished the capsaicin-induced increases in the IgE and EPO levels in the BAL fluid. The Cs-4 treatment (123 and 375 mg/kg) significantly decreased the IgE (36–66%) and EPO (37–64%) levels in the BAL fluid of capsaicin-challenged rats, when compared with the challenged and untreated controls.

### 2.13. Cs-4 Treatment Suppressed the Capsaicin-Induced Increases in the IL-4, IL-5, and IL-13 Levels in Rat Lung Tissue

**Figure 10:**
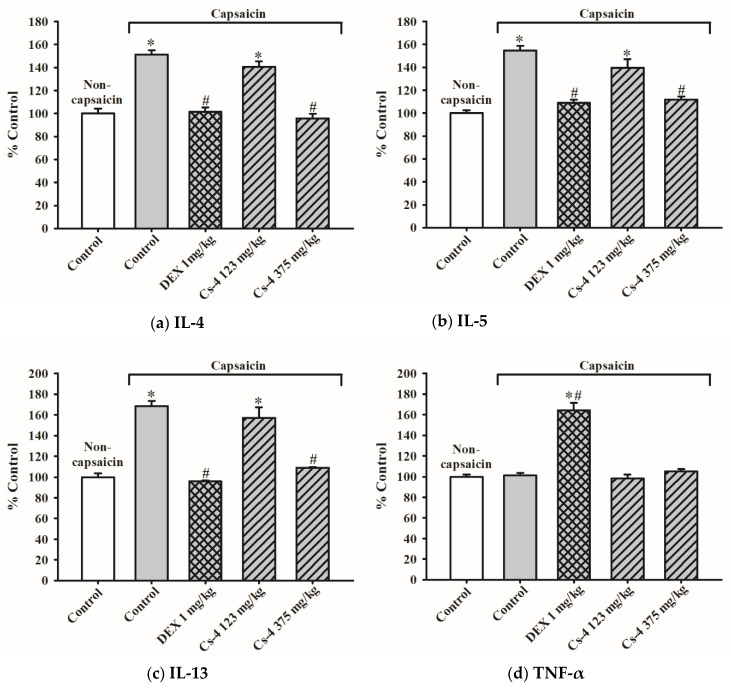

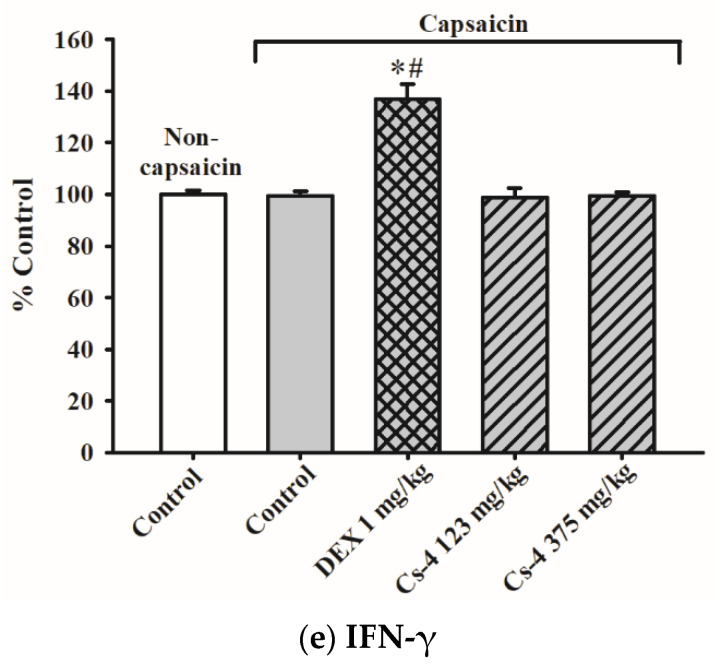
The effect of Cs-4 on various cytokine levels in the lung tissues of capsaicin-challenged rats. Data were expressed as the control percent of noncapsaicin rats, with the control values being (**a**) 367.6 ± 15.9 pg/mL (interleukin (IL)-4), (**b**) 368.1 ± 9.22 (IL-5), (**c**) 18.7 ± 0.67 pg/mL (IL-13), (**d**) 354.8 ± 7.39 pg/mL (tumor necrosis factor (TNF)-α), and (**e**) 745.6 ± 10.37 (interferon (IFN)-γ). Each bar represents the mean ± S.E.M., with *n* = 8. * Significantly different from the noncapsaicin control group. # Significantly different from the capsaicin control group.

Figure 10 shows the effects of Cs-4 on various cytokine levels in the lung tissue of capsaicin-challenged rats. The capsaicin challenge caused increases in IL-4 (51%), IL-5 (55%), and IL-13 (68%) in rat lung tissues when compared with the unchallenged controls. No detectable changes in the tumor necrosis factor (TNF)-α and interferon (IFN)-γ were observed. The DEX treatment (1 mg/kg) completely suppressed capsaicin-induced increases in IL-4, IL-5, and IL-13 levels in rat lung tissues and significantly increased the levels of TNF-α (64%) and IFN-γ (34%), when compared with both the unchallenged control and the challenged/untreated controls. The Cs-4 treatment almost completely suppressed the capsaicin-induced increases in IL-4, IL-5, and IL-13 levels in rat lung tissues at a dose of 375 mg/kg.

## 3. Discussion

In this study, animal models of allergic rhinitis and asthma were adopted to investigate the effects of Cs-4 treatments on nasal and airway allergies. Firstly, a mouse model of allergic rhinitis was produced by OVA sensitization followed by an OVA challenge, which resulted in nasal symptoms (nose rubbing and sneezing) and increases in IgE/OVA-IgE, as well as IL-4/IL-13 levels in the nasal fluid. The treatment with DEX (1 mg/kg × 10 days, before challenge) largely abrogated the nasal symptoms and completely suppressed the increases in IgE/OVA-IgE and IL-4/IL-13 levels in the nasal fluid in challenged mice. Daily oral treatments with Cs-4 (250 and 750 mg/kg × 10 days) also suppressed the nasal symptoms induced in OVA-sensitized and challenged mice. This inhibition was associated with reductions in IgE/OVA-IgE and IL-4/IL-13 levels in the nasal fluid. Additionally, Cs-4, as observed in the present study, suppressed the C48/80-induced histamine release from rat RPMC in vitro. Cytokines, such as IL-4 and IL-13, produced by Th2 play an important role in the induction and the sustaining of allergic responses [14]. IL-4 and IL-13 drive the immunoglobulin class switch and antigen-specific IgE-induced production of plasma cells [15]. These IgE antibodies bind with high affinity to Fc receptors on mast cells [16,17]. Allergens can induce mast cell activation and granule/histamine release from these cells. An immediate allergic response, which can lead to nasal symptoms, occurs within minutes after antigen exposure [18,19,20,21]. The ameliorative effect of DEX and Cs-4 treatments on AR is likely mediated by modulating the proinflammatory response triggered by an allergen.

Secondly, a rat model of asthma was produced by a neonatal capsaicin challenge, which was characterized by increases in the splenic index, cutaneous lesion score, airway responsiveness (i.e., tracheal and bronchial rings to Mch-induced contractions), and scratching behavior. Capsaicin-challenged rats showed increases in plasma IgE levels, as well as IgE and EPO levels, in the BAL fluid. Th2 cytokine (IL-4, IL-5, and IL-13) levels in lung tissues were also increased. The DEX treatment (1 mg/kg × 20 days following weaning) reduced the splenic index in capsaicin-challenged rats, but it also caused a reduction in body weight. Even though the DEX treatment did not produce any detectable effects on cutaneous skin lesions or airway responsiveness, it completely suppressed the stimulation of scratching behavior in capsaicin-challenged rats. Besides, the DEX treatment inhibited the capsaicin-induced increases in proinflammatory/inflammatory marker levels in the plasma and BAL fluid, as well as in rat lung tissues. While the capsaicin challenge did not change the levels of TNF-α and IFN-γ (Type 1 T helper cell (Th1) cytokines) in lung tissues, the DEX treatment caused significant increases. While the Cs-4 treatments (123 and 375 mg/kg × 20 days) did not change the body weights of the capsaicin-challenged rats, it did reduce the splenic index and lowered the cutaneous lesion scores. Both the decrease in airway responsiveness and amelioration of the scratching behavior afforded by the Cs-4 treatments were associated with reductions in plasma and BAL fluid proinflammatory/inflammatory marker levels in capsaicin-challenged rats. However, while the Cs-4 treatments completely suppressed increases in the cytokine (IL-4, IL-5, and IL-13) levels in rat lung tissues, it did not affect the levels of TNF-α or IFN-γ. The current literature considers that a skewed ratio of Th2 cytokines to Th1 cytokines indicates allergic inflammation. Th2 cell cytokines contribute to an allergic reaction, while Th1 cytokines contribute to nonallergic inflammation [22]. Th2 cytokines have been shown to be involved in the pathogenesis of asthma [22,23]. As such, the beneficial effect of DEX and Cs-4 treatments on asthma may be mediated by immunosuppressive actions on the production of Th2 cytokines. The differential effects of DEX and Cs-4 on skin lesions and airway responsiveness in capsaicin-challenged rats may be related to their distinct modes of action in immunomodulation. It has been shown that a reduced production of IFN-γ by the T cells of asthmatic patients correlates with disease severity [24,25] and that TNF-α is associated with the pathogenesis of asthma [23]. In this regard, the DEX-induced increase in IFN-γ in rat lung tissues, as observed in the present study, may be related to its preventive effect on asthma. DEX is a synthetic glucocorticoid that has anti-inflammatory and immunosuppressant effects and is used in several conditions, such as asthma and severe allergy [26]. Clinical studies have shown that patients receiving DEX, either at a high dose or for a long time, might develop several side effects, such as hyperglycemia, weight change, or osteoporosis, due to its in vivo nonselective actions [27]. The observation in our study is consistent with the weight reduction effect of the long-term DEX treatment in rats [28].

Consistent with the anti-AR and anti-asthmatic effects of Cs-4, it has been shown that a formulation of *Cordyceps militaris* produces an ameliorative effect in mouse model OVA-induced AR [29]. Besides, the *Cordyceps militaris* mycelium extract was found to modulate airway inflammation induced by OVA sensitization in mice [30]. In particular, a Cordyceps polysaccharide was shown to reduce the extent of airway inflammation in an OVA-induced mouse model of asthma [31]. An ultra-performance liquid chromatography coupled with ultraviolet detection (UPLC-UV) quantitative analysis indicated that Cs-4 contains adenine, adenosine, and cordycepin as the major constituents. In this regard, adenine has been demonstrated to inhibit IgE/antigen-induced degranulation and TNF-α release in mast cells [32]. The mechanism underlying the antiallergic effect of adenine involves the inhibition of Syk-mediated signal transduction and IκB kinase-mediated degranulation [32]. Adenosine has been reported to reduce inflammation by inhibiting leukocyte recruitment; adenosine also prevents stimulated neutrophils from adhering to vascular endothelium, as well as preventing neutrophil-mediated injury to the endothelium [33]. Cordycepin has been shown to exert anti-asthmatic activity in mice, which involves the inhibition of Th2-type responses through the suppression of the p38-MAPK and NF-κB signaling pathways [34]. Therefore, the anti-AR and anti-asthmatic effects of Cs-4 may be contributed to by the above-mentioned constituents.

In conclusion, our results suggest that Cs-4 (an extract of Cordyceps mycelium cultures) has the potential to alleviate immune hypersensitivity reactions in AR and asthma. When compared in human-equivalent doses, the long-term treatments with Cs-4, unlike that of DEX, which caused a body weight reduction in rats, produced undetectable side effects. The isolation of active components from Cs-4 for further investigation is warranted to elucidate the molecular mechanisms underlying its ameliorative effects on AR and asthma.

## 4. Materials and Methods

All animal experimental protocols were approved by the Animal Ethics Committee of the Hong Kong University of Science & Technology (Protocol number: 2018015).

### 4.1. Herbal Drug Preparation and UPLC-UV Analysis

Cs-4 extract, which is a commercial health product, was manufactured and supplied by Royal Medic Group Limited (Hong Kong SAR, China), with the contents of adenine, adenosine, and cordycepin being determined by UPLC-UV quantitative analysis. Briefly, Cs-4 powder was extracted by 25 mL of a mixture of water and ethanol (1:1, *v/v*). The mixture was sonicated for 30 min and then centrifuged at 3000× *g* at room temperature. The resultant supernatant was concentrated to 5 mL by rotary evaporation at 60 °C. The concentrated extract was reconstituted to a volume of 25 mL and gave a final concentration of 8 mg/mL. The Cs-4 extract (2 μL) was separated using a Waters ACQUITY UPLC system coupled with a photodiode array detector (Waters Corporation, Milford, MA, USA). A mobile phase consisting of water (solvent A) and acetonitrile (solvent B) was applied for programmed gradient elution with varying concentrations of A and B with time (0 min: 100% A and 0% B, 10 min: 97% A and 3% B, 18 min: 80% A and 20% B, and 21–24 min: 100% A) on a Waters ACQUITY UPLC BEH C18 column (130 Å, 1.7 μm, 2.1 mm × 100 mm) (Waters Corporation, Milford, MA, USA) at a flow rate of 0.25 mL/min and a temperature of 40 °C. The detection wavelength was set at 261 nm. The contents of adenine, adenosine, and cordycepin in the Cs-4 extract fraction were determined using a calibration curve.

### 4.2. The Mouse Model of Allergic Rhinitis

#### 4.2.1. Challenge and Treatment

Female adult Balb/c mice (~8 weeks of age; 15–20 g) were randomly assigned to 5 groups, with 10 animals in each group. CS-4 extract (at daily doses of 250 or 750 mg/kg; the lower one was a human-equivalent dose), DEX (1 mg/kg; in the range of a human-equivalent dose, obtained from Sigma-Aldrich, St. Louis, MO, USA), or distilled water was administered intragastrically by gavage for 10 daily doses. Mice were sensitized by the intraperitoneal administration of 200 µL of a mixture of 0.5-mg/mL ovalbumin (OVA; Sigma-Aldrich, St. Louis, MO, USA) and 20-mg/mL aluminum hydroxide (Alum) in phosphate-buffered saline (PBS-A) or PBS-A on days 0, 7, and 14. Seven days after the last sensitization (i.e., on day 21), mice were challenged intranasally with 10 µL of 25-mg/mL OVA in PBS-A through each nostril for 10 consecutive days (days 21–30). Non-OVA mice were treated with PBS-A only [35,36,37,38]. From day 21 to day 30, mice were intragastrically administered distilled water, Cs-4, or DEX 30 min before OVA challenge.

#### 4.2.2. Measurement of Nasal Symptoms

Nasal symptoms (sneezing and nose rubbing) were observed 1 min after the last OVA challenge. The number of sneezing and nose rubbings was counted for 20 min.

#### 4.2.3. Evaluation of IgE and Cytokines in NLF

Two hours after the last OVA challenge, mice were sacrificed. A ligation was made between the larynx and trachea using a thread. NLF was collected from the nostrils by perfusing 1-mL PBS-A into the larynx. The NLF collected was centrifuged at 10,000× *g* for 10 min at 4 °C to separate NLF from the cellular material. The supernatant was collected for cytokine measurement [39]. Levels of total IgE and OVA-specific IgE, as well as the level of cytokines (IL-4 and IL-13) released, were measured using ELISA kits (Cusabio, Houston, TX, USA).

### 4.3. RPMC Histamine Release

The effect of Cs-4 incubation on the mast cell histamine release was assessed using RPMC.

#### 4.3.1. RPMC Preparation

Sprague-Dawley rats (~2–6 months old) were anesthetized and intraperitoneally injected with 25 mL of Hank’s balanced salt solution containing 0.25-mM phenol red. The abdomen was gently massaged for about 60 s, and the fluid in the peritoneal cavity was aspirated. The retrieved cell suspension was centrifuged at 400× *g* for 5 min at 4 °C and washed with Gey’s solution to lyse red blood cells. The cell pellet was obtained after red blood cell lysis followed by centrifugation. The pellet was resuspended in 70% isotonic Percoll solution, which was then overlaid on a 4-(2-hydroxethyl)-1-piperazineethanesulfonic acid (HEPES)-tyrode buffer. The mixture was then centrifuged at 580× *g* for 15 min at 4 °C. The resultant mast cell pellet was resuspended in HEPES-tyrode buffer. The viability and purity of RPMC were assessed using Trypan blue and Toluidine blue staining, respectively [40,41].

#### 4.3.2. RPMC Degranulation

Purified RPMC were incubated with Cs-4 solution or HEPES-tyrode buffer for 15 min at 37 °C, then compound 48/80 (C48/80) at a final concentration at 1 mg/mL was added, and the preparation was incubated for 15 min. The reaction was stopped by chilling on ice for 10 min. The supernatant was collected by centrifugation at 20,000× *g* for 5 min at 4 °C. The cell pellet was lysed with 0.1% Triton X. The degree of degranulation was determined by measuring the release of β-hexosaminidase, a reliable indicator of mast cell degranulation [20]. The percentage of β-hexosaminidase release = absorbance of supernatant/(absorbance of supernatant + absorbance of lysate) × 100.

#### 4.3.3. RPMC Histamine Release

Purified RPMC were incubated with Cs-4 extract or HEPES-tyrode buffer for 15 min at 37 °C; then, C48/80 at a final concentration of 1 mg/mL was added, and the mixture incubated for 15 min. The reaction was stopped by chilling on ice for 10 min. The supernatant was collected by centrifugation at 20,000× *g* for 5 min at 4 °C. The pellet was lysed with water, and the solution was heated at 100 °C for 10 min. Histamine levels in the supernatant and lysate samples were measured using Elisa kits (Abcam, Cambridge, MA, USA) [42,43,44,45]. The histamine release was estimated as a percentage of the total histamine (both intracellular and extracellular) release.

### 4.4. The Rat Model of Asthma

A recent study has demonstrated that treating neonatal Sprague Dawley rats with capsaicin can result in an asthma-like phenotype, as evidenced by changes in the IgE levels; an increased number of eosinophils in the BAL fluid; and the increased mRNA expression of Th2 cytokines (such as IL-4, IL-5, and IL-13) in the lung tissue, as well as decreased airway resistance and compliance [46].

#### 4.4.1. Experimental Design

Sprague Dawley rats (~8 weeks of age; 15–20 g) were randomly assigned to 5 groups, with 10 animals in each group. Distilled water, Cs-4 (daily dose of 123 or 375 mg/kg; the lower one was a human-equivalent dose), or DEX (1 mg/kg; in the range of a human-equivalent dose) was administered intragastrically 5 days per week for 20 doses following weaning.

#### 4.4.2. Neonatal Capsaicin Treatment

Pregnant Sprague Dawley rats were obtained approximately 5 days before parturition. Within 24 h after birth, pups were randomly selected and injected subcutaneously with capsaicin (75 mg/kg; 50-mg capsaicin dissolved in 10 mL of a solution containing Tween 20:ethanol:saline in a ratio of 1:1:8 (*v/v*), while some pups received a vehicle and served as controls. All animals were kept in a room with a 12-h light-dark cycle at 22–25 °C, with free access to food and water. The pups were weaned at postnatal week 3, and only the male pups were used in the experiment [47].

#### 4.4.3. Scratching Behavior

Two hours after the last dose of treatment, rats were observed for behavioral changes. Briefly, rats were placed in separate cages for 10 min of adaptation. The behavior of the rats was then observed and recorded using a digital camera (TG-870, Olympus, Tokyo, Japan) for 29 min. The video files were played back on a computer, and the number of scratches counted by the experimenter. Nose rubbing and the motor behavior in which the hind paw moved up to the body and touched the skin a least once was taken as one count.

#### 4.4.4. Evaluation of Cutaneous Lesions

Cutaneous lesions were observed and assessed by scoring, as shown in Table 4. The skin conditions in the three regions (face, ears, and body) were subjected to scoring. The sum of the scores in the three regions was used as the dermatitis score for each rat.

#### 4.4.5. Body and Spleen Weight

Twenty-four hours after the last treatment, rats were anesthetized by an intraperitoneal injection of ketamine (80 mg/kg)/xylazine (5 mg/kg) dissolved in saline. The body weight was determined, and the spleen was dissected and weighed. The splenic index was estimated by the ratio of spleen weight (g)/body weight (g).

#### 4.4.6. Evaluation of AHR in Vitro

AHR following the methacholine challenge was measured in isolated tracheal and bronchial rings in vitro. Briefly, rats were anesthetized by intraperitoneal injection with a ketamine (80 mg/kg)/xylazine (5 mg/kg) mixture in saline. The thorax was opened, and 10-mL prewarmed Dulbecco’s Modified Eagle’s Medium (DMEM) with 1% low-melt agarose was injected into the lungs through the trachea. Lungs, trachea, and bronchi were filled with the medium, and a thread was used to tie the upper part of the trachea. Trachea and lungs were removed en bloc. The isolated lungs and the trachea were placed at 4 °C for 30 min. One or two rings of trachea and bronchi were dissected from the lung and put into prewarmed DMEM. Samples were incubated at 37 °C in a humidified atmosphere of 5% CO_2_ and 95% air overnight to dissolve the agarose. After the incubation, samples were washed with prewarmed DMEM, and the airway response of the tracheal and bronchial rings to increasing concentrations of Mch (Santa Cruz Biotechnology, Dallas, TX, USA) (from 10–9 to 10–0.5 M, respectively) was evaluated. Rings were incubated with a given concentration of Mch (starting with a low concentration) for 10 min at 37 °C, followed by observation under light microscopy and the image captured. The rings were sequentially challenged with six different concentrations of Mch, from low to high. The area of the lumen of each ring was measured in terms of the number of pixels using software ImageJ (LOCI). The initial percentage of the lumen size of each ring was calculated, and a graph plotting the percentage of the initial lumen size against a log molar concentration of Mch was drawn. The concentration of Mch at the one-half maximal decrease in the initial lumen size of the ring was estimated using the software GraphPad Prism6 (GraphPad Software, San Diego, CA, USA). Data were expressed as the mean ± standard error of the mean (S.E.M.). Data were analyzed by one-way ANOVA using SPSS statistical software version17 (IBM SPSS). Significant differences between the two groups were determined by the least significant difference at *p* < 0.05 [15,48,49].

#### 4.4.7. Biochemical Analyses

##### Sampling of Lung Tissues and Bronchoalveolar Lavage

Rats were anesthetized with a ketamine (80 mg/kg)/xylazine (5 mg/kg) mixture in saline by intraperitoneal injection 24 h after the last treatment. Blood was collected via cardiac puncture. The airways were cannulated with a syringe needle and rinsed with PBS-A without Ca^2+^ or Mg^2+^ (PBS-A). The fluid collected from the lavage was defined as the BAL fluid. Both lobes of the lung were acquired; then, a 20% lung homogenate was prepared with lysis buffer containing 0.5% Triton X-100, 150-mM NaCl, 15-mM Tris, 1mM-CaCl_2_, and 1mM-MgCl_2_, pH 7.4. The lung homogenates were then centrifuged at 10,000× *g* for 15 min at 4 °C. Supernatants were collected and kept in −80 °C before the assay [50].

##### IgE Levels

IgE levels in the plasma and in the supernatant produced from the BAL fluid were measured using a rat IgE ELISA kit (Cusabio, Houston, TX, USA) [46].

##### Th1/Th2 Cytokine Levels

The levels of IL-4, IL-5, IL-13, TNF-α, and IFN-γ in supernatants obtained from lung homogenates were measured using ELISA kits (Cusabio, Houston, TX, USA and Thermo Fisher Scientific, Waltham, MA, USA) [51].

##### Eosinophil Infiltration in the BAL Fluid

The extent of eosinophil infiltration was indirectly assessed by measuring the EPO level in the BAL fluid. The BAL fluid was centrifuged 10,000× *g* for 10 min at 4 °C, and the supernatant was collected for the measurement of EPO using ELISA kits (Cusabio, Houston, TX, USA).

### 4.5. Statistical Analysis

Unless otherwise specified, data were analyzed by one-way ANOVA using SPSS statistical software version17 (IBM SPSS). Significant differences between groups were determined by Tukey’s test with *p* < 0.05.

## Figures and Tables

**Table 1:** The effects of Cs-4 on the body weights and splenic index of capsaicin-challenged rats.

	Group	Body Weight (g)	Splenic Index
Noncapsaicin	Control	274 ± 4.04	0.00271 ± 0.00004
Capsaicin	Control	267 ± 6.39	0.00323 ± 0.00015 *
	DEX (1 mg/kg)	108 ± 2.48 *^#^	0.00178 ± 0.00007 *^#^
	Cs-4 (123 mg/kg)	269 ± 4.77	0.00323 ± 0.00011 *
	Cs-4 (375 mg/kg)	275 ± 5.56	0.00282 ± 0.00008 ^#^

* Significantly different from the noncapsaicin control group. # Significantly different from the capsaicin control group. DEX: dexamethasone.

**Table 2:** The effects of Cs-4 on the cutaneous lesions of capsaicin-challenged rats.

	Group	Score
Noncapsaicin	Control	0.150 ± 0.105
Capsaicin	Control	3.104 ± 0.148 *
	DEX (1 mg/kg)	2.167 ± 0.474 *
	Cs-4 (123 mg/kg)	2.462 ± 0.798 *
	Cs-4 (375 mg/kg)	0.952 ± 0.422 ^#^

* Significantly different from the noncapsaicin control group. # Significantly different from the capsaicin control group.

**Table 3:** The effect of Cs-4 on the airway responsiveness (AHR) in capsaicin-challenged rats in vitro. AHR was estimated by the minimum inhibitory concentration (EC_50_) of methacholine (Mch)-induced contractions, which were assessed by measuring the lumen area of the tracheal or bronchial rings. Values given are mean ± SEM.

	Group	Tracheal Ring	Bronchial Ring
		EC_50_ (μM)	EC_50_ (μM)
Noncapsaicin	Control	52.20 ± 24.97	15.39 ± 6.92
Capsaicin	Control	0.68 ± 0.38 *	0.61 ± 0.16 *
	DEX (1 mg/kg)	7.68 ± 7.02	0.90 ± 0.06
	Cs-4 (123 mg/kg)	24.84 ± 13.17^#^	24.00 ± 10.54 ^#^
	Cs-4 (375 mg/kg)	46.11 ± 23.26^#^	34.34 ± 17.88 ^#^

* Significantly different from the noncapsaicin control group. # Significantly different from the capsaicin control group.

**Table 4:** Evaluation of the cutaneous lesions.

Region	Score	Skin Condition
Face	0	Normal
	1	Wispy fur
	2	Alopecia and flare
	3	Bleeding or ulcerative lesion
Ears	0	Normal
	1	Flare
	2	Bleeding
	3	Loss of part of the ear
Body	0	Normal
	1	Wispy fur
	2	Alopecia and flare
	3	Bleeding or ulcerative lesion

## Abbreviations

AHR	Airway responsiveness
AR	Allergic rhinitis
BAL	Bronchoalveolar lavage
DEX	Dexamethasone
DMEM	Dulbecco’s Modified Eagle’s Medium
EPO	Eosinophil peroxidase
HEPES	4-(2-hyroxyethyl)-1-piperazineethanesulfonic acid
IgE	Immunoglobulin E
IL	Interleukin
IFN	Interferon
Mch	Methacholine
NLF	Nasal lavage fluid
OVA	Ovalbumin
PBS-A	Phosphate-buffer saline-A
RPMC	Rat peritoneal mast cell
Th1	Type I T helper cell
Th2	Type II T helper cell
TNF	Tumor necrosis factor
UPLC-UV	Ultra-Performance Liquid Chromatography coupled with Ultraviolet detection
